# Scoping review protocol for mapping policies on vaccine research, development and manufacturing in Africa

**DOI:** 10.12688/openresafrica.16549.1

**Published:** 2026-05-21

**Authors:** Adebisi Adenipekun, Eli Harriss, Uzma Alam, Samantha Vanderslott, Alice Norton

**Affiliations:** 1 Policy and Practice Research Group, Pandemic Sciences Institute, University of Oxford, Oxford, England, OX3 7DQ, UK; 2 Bodleian Health Care Libraries, University of Oxford, Oxford, England, OX3 9DU, UK; 3 Science For Africa Foundation, Nairobi, Nairobi County, Kenya

**Keywords:** Vaccine R&D, vaccine production, vaccine manufacturing, policy ecosystem, Africa

## Abstract

**Introduction:**

Despite the record-breaking speed of developing and manufacturing COVID-19 vaccines, there was a limited production capacity to promptly and equitably meet vaccine demands everywhere in the world. This, along with other factors such as national politics, economic power, and geopolitics, led to vaccine nationalism, in which some national governments prioritise access to vaccines for their citizens at the expense of global solidarity for equitable distribution.

Therefore, to secure Africa’s future access to vaccines, the African Union has announced a bold vision to produce at least 60 percent of the vaccines consumed locally in Africa by 2040, as part of the broader health security and future pandemic response agenda. Achieving this vision requires proactive policy coherence and coordination across the continent to prevent duplication of efforts, waste and unhealthy competition.

Consequently, this review aims to map, assess and compare the content of national, regional, and continental-level policies for local vaccine production in Africa, generating actionable insights to inform coordinated strategies and advance the AU’s 2040 vaccine production goal.

**Methods and analysis:**

The review will be conducted following the Arksey and O’Malley framework and in accordance with the Joanna Briggs Institute’s methodological guidance for scoping reviews. Reporting will adhere to the PRISMA-ScR guidelines. Both published and unpublished policy-related documents will be included by searching the following databases – Overton, Policy Commons, and ProQuest Policy File Index – as well as performing a Google search of official government and relevant institutions’ websites. The search will focus on Africa, using key terms relating to vaccine policies. Two independent reviewers will screen the retrieved documents in Covidence, and data extraction will be conducted using a pretested template in Microsoft Excel 2021.

**Conclusion:**

This scoping review does not require ethical approval. A comprehensive manuscript detailing the review’s findings will be published upon the study’s completion.

## Introduction

The COVID-19 pandemic revealed gaps in the efficiency and effectiveness of established norms governing global vaccine research and development (R&D), manufacturing, and supply chains serving global populations equitably. ^1^ Before the pandemic, vaccines were produced and supplied mainly from high-income countries ^2, 3^ and some emerging economies, ^4, 5^ despite the large volume of vaccine consumption driven by growing population and high burden of infectious diseases in low- and middle-income countries (LMICs). ^6– 8^ For example, as of 2022, Africa consumed approximately 1 billion vaccine doses, ^9^ accounting for about 25 percent of the global vaccine volume produced. ^8^ Whereas African manufacturers supply less than one percent of the continent’s total vaccine demand. ^9^

Despite the record-breaking speed of developing and manufacturing COVID-19 vaccines, production capacity was limited, largely concentrated in a few high-income countries, and was unable to promptly and equitably meet vaccine demand worldwide. ^10– 12^ The inadequate vaccine supply capacity, coupled with other factors such as local politics, triggered vaccine nationalism, described as a ‘my country first’ approach and focus on national interests through banning vaccine exports from producing countries or entering into supply agreements exclusively with manufacturers without considering demands for the most at-risk populations. ^13, 14^

Vaccine nationalism during the COVID-19 pandemic was observed among high-income countries (HICs), which procured significantly more vaccines than needed, thereby blocking LMICs and LICs from obtaining timely access to the vaccines during the pandemic. ^10^ Another manifestation of vaccine nationalism was vaccine hoarding. Countries like India, which, due to their lower production costs and economies of scale, produce at rates more affordable for the global South, banned COVID-19 vaccine exports to prioritise access for their citizens. ^15, 16^

One of the initiatives that is important and relevant to future epidemic and pandemic resilience and response in Africa is the call to expand global vaccine manufacturing capacity and develop regional capacity for vaccine production worldwide. ^10^ Sanjana et al. argued that leveraging countries with small populations for expanded vaccine production can help address the challenge of vaccine nationalism, as they can quickly meet their own needs and contribute to the global supply. However, of the twelve countries identified with both a small population and vaccine manufacturing capacity, seventy-five percent were from Europe, and none were from the African region. ^17^ Hence, this model could increase global production capacity, but does not guarantee prompt and equitable access in LMICs and LICs.

Specifically in Africa, the African Union (AU) and the Africa Centres for Disease Control and Prevention (Africa CDC), the autonomous health agency of the African Union established to support public health initiatives of Member States and strengthen the capacity of their public health institutions, have announced a bold vision of producing at least 60 percent of the vaccines consumed locally in Africa by 2040. ^9^

To achieve this, the Africa CDC has published a framework for action (FFA) for the Partnership for African Vaccine Manufacturing (PAVM). The PAVM FFA presents the key diagnostic findings for Africa’s vaccine manufacturing environment. It provided a clear pathway from current vaccine manufacturing on the continent to what is required to achieve the vision 2040 set by the AU and Africa CDC. ^9^

According to the framework, the challenges hindering Africa’s R&D efforts to transition to a fully integrated R&D value chain include insufficient and inappropriate R&D infrastructure for advancing and commercialising vaccine production. Vaccine production encompasses the end-to-end process of creating a vaccine, ranging from R&D to manufacturing, including large-scale production and all steps involved in quality and safety assurance and regulatory approval. ^18, 19^ Hence, among other strategic directions, the framework is set to develop an R&D coordinating platform and establish pilot current Good Manufacturing Practice (cGMP) batch manufacturing facilities across the continent.

For this study, I will focus on the vaccine R&D pillar of the framework, with a specific interest in the policy ecosystem, that is, the interaction of policies, actors, contexts and institutions, including emerging geopolitical shifts, that influence the development and implementation of policies for vaccine R&D and manufacturing in Africa. ^20^ The focus on vaccine R&D is important given its vital role in developing routine and future emergency vaccines. As the world returns to normal after the COVID-19 pandemic, commitment to vaccine R&D in Africa can help develop new vaccines, improve existing ones, create new combinations of existing vaccines or improve processes to ensure affordability and availability of vaccines on the continent.

There have been publications on the continental efforts to ramp up vaccine production in Africa, ^7, 8, 21^ including a recently published landscape analysis of the vaccine ecosystem in Africa by Catherine Fleck-Vidal et al. ^22^ Yet, there is a gap in the literature on how the policy ecosystems of different African countries create an enabling or inhibiting environment for local production that aligns with the continental agenda. A preliminary search of MEDLINE, the Cochrane Database of Systematic Reviews and *JBI Evidence Synthesis* was conducted. No current or underway systematic or scoping reviews on the topic were identified.

This scoping review aims to identify, map and compare the content of national, regional and continental-level policies on local vaccine production in Africa, generating actionable insights to inform coordinated strategies and advance the AU’s 2040 vaccine production goal.

This review aims to answer two broad questions. Firstly, what is the content of national, regional and continental-level policies for local vaccine production in African countries? Secondly, how do local vaccine production policies and national-level agendas align or misalign with the continental agenda and priorities?

## Protocol

### Inclusion criteria

Documents considered for inclusion in this study are policies, laws, and regulations related to vaccine production (R&D and manufacturing) in Africa. We will include policy white papers, reports, documents, and articles on vaccine production in Africa. Only the latest edition currently in force will be included for policy documents with different editions. This scoping review will cover relevant documents produced by international organisations such as the WHO and Gavi, as well as countries with bilateral ties to Africa as a continent or specific African nations. Articles on technology transfer or decentralised vaccine manufacturing in LMICs and LICs will be included only if they contain specific references to Africa or particular African countries. There will be no restrictions on the timeframe of included resources. All languages recognised as official in African countries, which can be translated into English using Google Translate, will be considered in this study.

### Types of sources

We will search the following databases for eligible policy documents: Overton, Policy Commons, and ProQuest Policy File Index.

These will be complemented by results from Google searches for the official government websites of the five regions and 55 African countries, as well as the websites of relevant institutions such as the Africa CDC, the African Union, AUDA NEPAD, and the WHO Africa Region.

### Framework

The review will follow the framework recommended by Arksey and O’Malley, which involves identifying the research question; selecting relevant studies; choosing studies; charting the data; and collating, summarising, and reporting the results. ^23^ It will also adhere to the Joanna Briggs Institute’s methodological guidance for conducting scoping reviews, ^24^ and the reporting will conform to the Preferred Reporting Items for Systematic reviews and Meta-Analyses extension for Scoping Reviews (PRISMA-ScR) guidelines. ^25^

### Search strategy

We aim to identify relevant published policy documents for this review. Working with an experienced librarian from the University of Oxford Bodleian Health Care Libraries, we developed a robust search strategy that will be implemented in three steps. First, a preliminary limited pilot search of Overton, as shown in Appendix I, provided as an extended data, ^26^ will be conducted using keywords such as vaccine production, vaccine research and development (R&D), vaccine manufacturing, policy, policy ecosystem, policy landscape, and policy environment.

Secondly, the words used in the titles and abstracts of the documents identified in the pilot search will inform the development of the full search strategy.

Lastly, the complete search strategy, including the identified keywords and index terms, will be revised to suit other databases and information sources, as shown in Appendix II of the extended data. ^26^

Due to limited resources, we will not review the bibliographies of the documents included in this policy mapping review.

### Study/Source of evidence selection

We will use EndNote version 20.6 (Bld 17174), a citation and reference management tool, to organise the selected documents and screen for duplicates. The remaining documents after deduplication will be exported into Covidence, a systematic review management platform, for the title and abstract screening, which will be a blinded process conducted by two screeners with a thorough understanding of the eligibility criteria.

Documents that pass the first screening will advance to the second screening stage, the full-text screening. Conflicts in the first and second screening rounds will be resolved by consensus or discussion with a third reviewer. We will use the PRISMA-ScR (Preferred Reporting Items for Systematic reviews and Meta-Analyses extension for Scoping Reviews) flow diagram to report the process of selecting sources.

### Data extraction

A pilot extraction sheet using Microsoft Excel 2021 will be created. The sheet will be adjusted as needed during the extraction process. As detailed in Appendix III of the extended data, ^26^ the information to be recorded includes the author(s), year of publication, place of publication, the document’s aims, and the methodology or approach used. Additional items to be extracted will be based on the vaccine production frameworks, covering the discovery, development, and delivery phases. We will also address the policy ecosystem, including policy contents, context, process, and actors.

### Data analysis and presentation

We will present the results of this scoping review using the vaccine production and policy ecosystem framework. We will use descriptive analysis to synthesise and present the types and sources of documents included in the scoping review. We will code the qualitative data into relevant categories and frameworks. We will use Microsoft Excel 2021 and NVivo, a qualitative data analysis software for data management and analysis.

## Conclusion

This scoping review does not require ethical approval. A detailed manuscript detailing the results of the review will be published upon completion of the study.

## Data Availability

There is no underlying data associated with this article. Figshare: Scoping review protocol: Search strategy, data extraction instrument and PRISMA-ScR checklist. https://doi.org/10.6084/m9.figshare.31949979.v1 (Adenipekun 2026). This project contains the following extended data:
• Appendix I: Pilot search strategy • Appendix II: Database search strategy • Appendix III: Data extraction instrument Data are available under the terms of the Creative Commons Attribution 4.0 International license (CC-BY 4.0). We will use the PRISMA-ScR (Preferred Reporting Items for Systematic reviews and Meta-Analyses extension for Scoping Reviews), available in the extended data ( https://doi.org/10.6084/m9.figshare.31949979), to report this study.
