# Mapping policy architecture for vaccine research, development and manufacturing in Africa: a scoping review protocol

**DOI:** 10.12688/openresafrica.16549.3

**Published:** 2026-10-06

**Authors:** Adebisi Adenipekun, Eli Harriss, Uzma Alam, Samantha Vanderslott, Alice Norton

**Affiliations:** 1 Policy and Practice Research Group, Pandemic Sciences Institute, University of Oxford, Oxford, England, OX3 7DQ, UK; 2 Bodleian Health Care Libraries, University of Oxford, Oxford, England, OX3 9DU, UK; 3 Science For Africa Foundation, Nairobi, Nairobi County, Kenya

**Keywords:** Vaccine R&D, vaccine production, vaccine manufacturing, policy ecosystem, Africa

## Abstract

**Introduction:**

Despite the unprecedented speed of COVID-19 vaccine development, manufacturing capacity remained geographically concentrated in high-income and some emerging economies and was insufficient to meet global demand promptly and equitably. This constrained production capacity, together with domestic political pressures and market power asymmetries, contributed to vaccine nationalism. Therefore, to secure Africa’s future access to vaccines, the African Union aims to produce at least 60 percent of the vaccines consumed locally in Africa by 2040, up from less than one percent, as part of the broader health security and future pandemic response agenda. Achieving this vision requires proactive policy coherence and coordination across the continent to prevent duplication of efforts, waste and unhealthy competition. Consequently, this review aims to map, assess and compare the content of national, regional, and continental-level policies for vaccine R&D and manufacturing in Africa, generating actionable insights to inform coordinated strategies and advance the AU’s 2040 vaccine manufacturing goal.

**Methods and analysis:**

The review will be conducted following the Arksey and O’Malley framework and in accordance with the Joanna Briggs Institute’s methodological guidance for scoping reviews. Reporting will adhere to the PRISMA-ScR guidelines. Policy instruments relevant to human vaccine R&D and manufacturing in Africa will be included as the primary unit of analysis. The sources of evidence are policy databases – Overton, Policy Commons, and ProQuest Policy File Index – and the official websites of relevant governments, regional and continental institutions. The search will focus on Africa, using key terms relating to vaccine policies. Two independent reviewers will screen the retrieved documents in Covidence, and data extraction will be conducted using a pretested template in Microsoft Excel 2021. This review does not require ethical approval.

**Conclusion:**

The results will contribute to the academic literature on vaccine security and the policy architecture for vaccine R&D and manufacturing in Africa.

## Introduction

The COVID-19 pandemic exposed structural weaknesses in global vaccine research and development (R&D), manufacturing, and supply arrangements, particularly with respect to equitable access across regions and income groups. ^1^ Before the pandemic, vaccine production was concentrated in high-income countries ^2^ ^,^ ^3^ and a small number of emerging economies, ^4^ ^,^ ^5^ despite substantial vaccine demand in low- and middle-income countries (LMICs). ^6^ ^–^ ^8^ For example, as of 2022, Africa consumed approximately 1 billion vaccine doses, ^9^ accounting for about 25 percent of the global vaccine volume produced. ^8^ Whereas African manufacturers supply less than one percent of the continent’s total vaccine demand. ^9^

Despite the unprecedented speed of COVID-19 vaccine development, manufacturing capacity remained geographically concentrated in high-income countries and some emerging manufacturing economies and was insufficient to meet global demand promptly and equitably. ^10^ ^–^ ^12^ This constrained production capacity, together with domestic political pressures and market power asymmetries, contributed to vaccine nationalism, described as a ‘my country first’ approach and focus on national interests through banning vaccine exports from producing countries or entering into supply agreements exclusively with manufacturers without considering demands for the most at-risk populations. ^13^ ^,^ ^14^

Vaccine nationalism during the COVID-19 pandemic was observed among high-income countries (HICs), which, although representing 16% of the global population, pre-ordered and secured at least 70% of the available COVID-19 vaccine doses for the five leading vaccine candidates in 2021, targeting enough doses to vaccinate most of their countries’ adult populations, thereby blocking LMICs and LICs from obtaining timely access to the vaccines for prioritised populations such as health-care workers and other high-risk populations. ^10^ Apart from the disproportionate advance procurement and domestic prioritisation of vaccine supplies by HICs, another manifestation of vaccine nationalism was temporary restriction of vaccine exports by producing countries and regions. For example, the United States (US), India and the European Union (EU) temporarily banned COVID-19 vaccine exports to prioritise access for their citizens. ^15^ ^,^ ^16^

One of the initiatives that is important and relevant to future epidemic and pandemic resilience and response in Africa is the call to expand global vaccine manufacturing capacity and develop regional capacity for vaccine production worldwide. ^10^ Sanjana et al. argued that leveraging countries with small populations for expanded vaccine production can help address the challenge of vaccine nationalism, as they can quickly meet their own needs and contribute to the global supply. However, of the twelve countries identified with both a small population and vaccine manufacturing capacity, seventy-five percent were from Europe, and none were from the African region. ^17^ Hence, this model could increase global manufacturing capacity, and could contribute to access in LMICs/LICs where sustainable manufacturing capacity can be developed, but the current geographic distribution does not guarantee prompt and equitable access in LMICs and LICs.

Specifically in Africa, the African Union (AU) and the Africa Centres for Disease Control and Prevention (Africa CDC), the autonomous health agency of the African Union established to support public health initiatives of Member States and strengthen the capacity of their public health institutions, have announced a bold vision of manufacturing at least 60 percent of the vaccines consumed locally in Africa by 2040. ^9^

To achieve this, the Africa CDC has published a framework for action (FFA) for the Partnership for African Vaccine Manufacturing (PAVM). The PAVM has been revised to the Platform for Harmonised African Health Manufacturing (PHAHM), reflecting the broader continental agenda for manufacturing of therapeutics, vaccines, diagnostics and medical devices. The PAVM FFA presents the key diagnostic findings for Africa’s vaccine manufacturing environment. It provided a clear pathway from current vaccine manufacturing on the continent to what is required to achieve the vision 2040 set by the AU and Africa CDC. ^9^

According to the framework, the challenges hindering Africa’s vaccine R&D efforts to transition to a fully integrated R&D value chain include insufficient and inappropriate R&D infrastructure for advancing and commercialising vaccine production. Vaccine production encompasses the end-to-end process of creating a vaccine, ranging from R&D to manufacturing, including large-scale production and all steps involved in quality and safety assurance and regulatory approval. ^18^ ^,^ ^19^ Hence, among other strategic directions, the framework is set to develop an R&D coordinating platform and establish pilot current Good Manufacturing Practice (cGMP) batch manufacturing facilities across the continent.

This study will focus on the end-to-end process of the vaccine production value chain, including vaccine R&D and manufacturing, with a specific interest in the policy architecture. Policy architecture refers to the structural blueprints, rules, and instruments and how they shape interactions among policies, actors, contexts and institutions that influence the development and implementation of policies for vaccine R&D and manufacturing in Africa. ^20^ As countries transition from the acute phase of the COVID-19 pandemic, commitment to vaccine R&D and manufacturing in Africa can help develop new vaccines, improve existing ones, create new combinations of existing vaccines or improve processes to ensure affordability and availability of vaccines on the continent.

There have been publications on the continental efforts to ramp up vaccine production in Africa, ^7^ ^,^ ^8^ ^,^ ^21^ including a published landscape analysis of the vaccine ecosystem in Africa by Catherine Fleck-Vidal et al. ^22^ In complementary but distinct reviews, Amaechi et al. focus on country-level manufacturing readiness, synthesising relevant frameworks, indicator sets, and policies to inform multidomain readiness assessment, while Ataguba et al rapidly synthesised policy, legal, governance and investment frameworks in relation to vaccine R&D capacity, manufacturing and equitable access. (Amaechi et al 2025 and Ataguba 2026). However, while Amaechi et al. sought to ask and objectively measure whether African countries are ready for human vaccine manufacturing, and Ataguba et al. asked what frameworks influence capacity and equity, this scoping review asks whether Africa has a coherent policy architecture capable of connecting vaccine research to sustainable local manufacturing. Noting that neither Amaechi et al. & Ataguba et al’s work was designed primarily as a comparative analysis of formal African policy instruments themselves across the vaccine R&D-to-manufacturing continuum, this review will take policy instruments as its primary unit of analysis and examine policy content, governance arrangements, implementation orientation, and horizontal and vertical policy coherence across national, regional and continental levels.

This scoping review aims to identify, map and compare the content of national, regional and continental-level policies on vaccine R&D and manufacturing in Africa, generating actionable insights to inform coordinated strategies and advance the AU’s 2040 vaccine manufacturing goal.

The primary question for this review is: How are national, regional and continental policy instruments for human vaccine R&D and manufacturing structured across Africa? The review aims to answer four secondary questions. Firstly, what formal policy instruments relevant to human vaccine R&D and manufacturing exist at national, regional and continental levels? Second, what is the content of these policies in terms of objectives, enforcement mechanisms, responsible institutions, financing provisions, targets, timelines, monitoring and evaluation approaches, and implementation mechanisms? Thirdly, which stages of the vaccine R&D-to-manufacturing continuum do these policies address? Lastly, where do national policies align or misalign with national, regional, and continental policies and priorities?

## Protocol

### Inclusion criteria

This scoping review uses policy instruments relevant to human vaccine R&D and manufacturing in Africa as its primary unit of analysis. As shown in Table 1, the documents considered for inclusion are policy instruments defined as authoritative documents issued or formally endorsed by a governmental, intergovernmental, or recognised regional or continental governance body, such as the African Union, that establish goals, rules, commitments, responsibilities, and incentives for human vaccine R&D or manufacturing in Africa. This includes legal instruments such as legislation, regulations, Acts, and other legally binding documents; strategic policy instruments such as strategic roadmaps, action plans, and national strategies; industrial instruments such as industrial policies, local procurement policies, and trade policies; scientific instruments such as research policies, innovation strategies, and R&D funding policies; and relevant enabling policy instruments such as regulatory harmonisation strategies, workforce policies, infrastructure policies, technology transfer arrangements, and intellectual property (IP) policies.

**Table 1.** Decision rules for data sources inclusion and exclusion.

S/n	Criterion	Eligible (include)	Exclude	Example
1.	Geographical scope	Policy instrument concerns one or more African countries, an African regional body, or Africa at the continental level	Contains no substantive application to African countries/regions or continent	Include: African Union strategy document on vaccine manufacturing. Exclude: European Union strategy on vaccine manufacturing in Europe.
2.	International organisations with mandates including Africa	Document contains policy commitment, framework or programme, such as finance mechanisms, technology transfer arrangements, governance mechanisms, directly applicable to vaccine R&D or manufacturing in Africa or specific African countries	Global strategy not directly intended for African countries, regions or continent.	Include: Gavi’s African Vaccine Manufacturing Accelerator (AVMA). Exclude: WHO guidelines on the international packaging and shipping of vaccines.
3.	Human vaccines	Document addresses policies relevant to R&D, innovation or manufacturing of vaccines for human use	Pharmaceuticals or biologics broadly, without identifiable relevance to human vaccines. Veterinary vaccines are not eligible.	Include: National biotechnology strategy with specific provisions for human vaccine R&D or manufacturing. Exclude: National veterinary vaccine production policy.
4.	Formal policy instrument – primary unit of analysis	Document issued, adopted or endorsed by a government, ministry, regulator, regional body, continental institution that establishes objectives, rules, commitments, responsibilities or implementation mechanisms relevant to vaccine R&D and manufacturing	Documents with recommended policies without constituting an authoritative policy instrument	Include: African Union framework for action on vaccine manufacturing. Exclude: Academic commentary recommending or endorsing vaccine strategy
5.	National policies not labelled “vaccine policy”	Science, health, pharmaceutical, trade, innovation, or biotechnology policy with explicit and substantive provisions * on human vaccine R&D/manufacturing.	Mentions “vaccines” without a policy objective, commitment, intervention or implementation provision relating to vaccine R&D/manufacturing.	Include: National pharmaceutical policy with substantive content and commitment to domestic vaccine production. Exclude: National health policy focused on vaccination coverage only.
6.	Vaccine R&D	Document includes basic or applied research on vaccines, vaccine discovery, preclinical development, clinical trials, translational research or R&D infrastructure/capacity. This has to do with the upstream	Broadly addresses general health research or scientific R&D without identifiable vaccine component.	Include: National vaccine R&D strategy Exclude: University research policy with no substantive provisions * on vaccine R&D
7.	Vaccine manufacturing	Document addresses antigen/drug-substance production, formulation, fill-and-finish, scale-up, and other manufacturing activities.	Focused only on vaccination delivery or cold chain operations after manufacture.	Include: National local vaccine manufacturing plan. Exclude: Routine immunisation cold-chain plan with no production component.
8.	Regulatory policy	Policy instruments directly related to vaccine R&D, clinical development, technology transfer or local manufacturing.	Regulations on vaccine administration and pharmacovigilance without direct link to vaccine R&D/manufacturing.	Include: Policy strengthening regulatory pathways for locally developed vaccines. Exclude: Vaccination-site safety guidance.
9.	Technology transfer	Policy to govern, guide, facilitate or commit to technology/know-how transfer to or with Africa for vaccine R&D/manufacturing.	Technology transfer is discussed in the context of global access to vaccines, without specific application to African countries or the continent.	Include: WHO’s mRNA technology-transfer programme in Africa. Exclude: A generic article on decentralised manufacturing in LMICs with no specific African policy content.
10.	Intellectual property	Policy instruments on patents, licensing and IP provisions regarding the African vaccine R&D/manufacturing architecture.	The document discusses TRIPS/IP generally, without a substantive provision connecting it to African vaccine R&D/manufacturing.	Include: National policy on use of licensing mechanisms to facilitate local vaccine manufacturing. Exclude: Report on pharmaceutical patents in LMICs
11.	Economic/trade/finance/Investment	Policy instruments on Africa inter- and intra-continental trade agreements relating to vaccine R&D/manufacturing. Also includes financing, incentives, subsidies, guarantees, tax measures and other investment mechanisms for vaccine R&D/manufacturing.	Report of investments without detailing or describing a relevant policy mechanism/approach	Include: National tax policy to incentivise local vaccine manufacturing. Exclude: Report estimating capital requirement for vaccine manufacturing in Africa without providing an authoritative policy pathway to financing.
12.	Workforce/skills	Documents contain explicit measures to develop the personnel needed for vaccine R&D/manufacturing.	General health-workforce training or retention in Africa without specificity on vaccine R&D/manufacturing.	Include: Biotechnology workforce strategy specifiying vaccine R&D or GMP skills. Exclude: Document on immunisation training programme for nurses or community extension workers.
13.	Market shaping/procurement	Policy instruments to ensure vaccine R&D and manufacturing are sustainable in Africa.	Market shaping arrangements to ensure vaccine supply without a local-production linkage.	Include: Policy on local-preference procurement for African-manufactured vaccines. Exclude: Routing national vaccine procurement schedule.
14.	Infrastructure	Policy instruments that address inputs, facilities, equipment, utilities or supply chains needed for vaccine R&D/manufacturing.	Focuses only on supply chain and distribution logistics.	Include: Industrial policy supporting local production of vaccine manufacturing inputs. Exclude: Last-mile vaccine distribution policy
15.	Policy status	Policy instruments formally adopted, enacted, approved or otherwise officially in force.	Unverifiable proposals, media speculations or informal recommendations represented as government policy.	Include: Gazetted Act Exclude: Media report announcing the Act
16.	Superseded policy versions	Current versions are eligible as the primary data source, while earlier versions are retained where needed to analyse substantive policy evolution over time.	Exclude duplicate/administrative versions that add no substantive policy information.	Include: he 2018 and 2024 strategies when comparing how policy changed over time. However, data extraction for analysis uses the 2024 version. Exclude: Drafts of the 2024 version available online.
17.	Publication date	No date restrictions on eligible policy instruments. The adoption, amendment and expiry/supersession dates will be recorded.	Not applicable	Include: All eligible policy instruments irrespective of dates. Note the latest version differently from superseded versions.
18.	Language	Eligible policy instruments available in English	Policy instruments not translated to English by the government or institution which published it.	Include: Relevant WHO Afro document published in English and other official African languages. Exclude: Policy instruments that the author(s) have not published English version.
19.	Accessibilty	Full policy text, authoritative archived version, or sufficiently complete official text is obtainable for content analysis.	Broken link, citation, news summary and policy’s substantive content cannot be established.	Include: Government policy available as a full text. Exclude: Government webpage announcing a policy without access to the full text (except if it is accessible from other sources/databases).
20.	Duplicate documents	Retain one authoritative version and link duplicate copies to the same policy record.	Exclude identical copies hosted by multiple organisations as separate policies/documents	The same AU policy available on AU and Africa CDC websites.

* A substantive provision is defined as an identifiable objective, rule, commitment, intervention, resource allocation, institutional responsibility, incentive, target or implementation mechanism. A mere mention of vaccine R&D or manufacturing will not be sufficient for inclusion.

In terms of geographical scope, eligible primary sources are policy instruments issued or formally endorsed by African national governments, African regional economic communities, the African Union or its agencies, and other recognised African intergovernmental bodies. Policy instruments issued by non-African/international organisations will be eligible only where they establish an explicit policy commitment, governance arrangement, technology-transfer mechanism or financing mechanism applicable to vaccine R&D or manufacturing in Africa. The latest edition of a policy instrument currently in force will be the primary data source; however, superseded policies will be reviewed to track policy evolution. External policy instruments, including documents produced by international organisations such as the WHO and Gavi, are only eligible if they establish a policy commitment, governance mechanism, technology-transfer arrangement or financing mechanism explicitly applicable to vaccine R&D or manufacturing in Africa. Peer-reviewed articles, commentaries, evaluations and reports describing vaccine R&D and manufacturing policy development and implementation will be used as secondary contextual evidence and will not be included as primary sources for data extraction. There will be no restrictions on the timeframe of included resources. This review will include only policy instruments available in English.

### Types of sources

Policy instruments for this scoping review will be obtained from three broad categories of sources.

1. **Policy databases:** The following databases were searched for eligible policy instruments: Overton, Policy Commons, and ProQuest Policy File Index. The detailed search strategy, including the search string for the pilot search, is included in Appendix I (Adenipekun 2026).
2. **Continental and regional institutions search:** The website of African Union, Africa CDC, AUDA-NEPAD, AfDB, WHO Africa Region and RECs would be searched for eligible documents.
3. **Official national governments search:** Websites of relevant national ministries, departments and agencies (MDAs) would be searched systematically by screening the first 10 pages of Google search results for eligible documents or links to appropriate websites to download the documents. An example Google search input is “Vaccine R&D and manufacturing related policies in Nigeria”; the country name would be replaced with other African countries when searching other countries on the continent, while “vaccine R&D and manufacturing” would be replaced with “regulatory”; “technology transfer”; “intellectual property”; “economic and trade”; “workforce and skills”; and “Local procurement” to capture the different dimensions of possible national and sub-national policy instruments relevant to the objectives of this scoping review. To ensure transparency and reproducibility, a detailed grey-literature search log will be used to document and report the country, website, search terms, date searched, results examined, documents retrieved, and the reason for inclusion/exclusion.
  The initial pilot search was conducted in October 2025, and the database search strategy was finalised in January 2026. The review and scoping of the policy database, continental and regional institutions, and national governments’ websites are ongoing and are projected to be complete by September 2026.

### Framework

The review will follow the framework recommended by Arksey and O’Malley, which involves identifying the research question; selecting relevant studies; choosing studies; charting the data; and collating, summarising, and reporting the results. ^23^ It will also adhere to the Joanna Briggs Institute’s methodological guidance for conducting scoping reviews, ^24^ and the reporting will conform to the Preferred Reporting Items for Systematic reviews and Meta-Analyses extension for Scoping Reviews (PRISMA-ScR) guidelines. ^25^

### Search strategy

Working with an experienced librarian from the University of Oxford Bodleian Health Care Libraries, a robust search strategy will be implemented in three steps for the policy database search. First, a preliminary limited pilot search of Overton, as shown in Appendix I, provided as an extended data, ^26^ will be conducted using keywords such as vaccine production, vaccine research and development (R&D), vaccine manufacturing, vaccine policy, vaccine policy architecture, vaccine policy ecosystem, vaccine policy landscape, and vaccine policy environment.

Secondly, the words used in the titles and abstracts of the documents identified in the pilot search will inform the development of the full search strategy.

Lastly, the complete search strategy, including the identified keywords and index terms, will be revised to suit other databases and information sources, as shown in Appendix II of the extended data. ^26^

Due to limited resources, review of the bibliographies of the documents included in this scoping review would not be done.

### Study/Source of evidence selection

EndNote version 20.6 (Bld 17174), a citation and reference management tool will be used to organise the selected documents and screen for duplicates. The remaining documents after deduplication will be exported into Covidence, a systematic review management platform, for title and abstract screening, which will be a blinded process conducted by two screeners with a thorough understanding of the eligibility criteria.

Documents that pass the first screening will advance to the second screening stage, the full-text screening. Conflicts in the first and second screening rounds will be resolved by consensus or discussion with a third reviewer. The PRISMA-ScR (Preferred Reporting Items for Systematic reviews and Meta-Analyses extension for Scoping Reviews) flow diagram will be used to report the process of selecting sources.

### Data extraction

A pilot extraction sheet using Microsoft Excel 2021 will be created. The sheet will be adjusted as needed during the extraction process. As detailed in Appendix III of the extended data, ^26^ the information to be recorded includes the author(s), year of publication, place of publication, the document’s aims, and the methodology or approach used. Additional items to be extracted will be based on the vaccine production frameworks, covering the discovery, development, and delivery phases. The tool will also address the policy architecture in terms of existing policies and their contents, context, process, and identified actors.

### Data analysis and presentation

Two complementary analytical frameworks will guide the analysis. First, policy instruments will be mapped across the vaccine R&D-to-manufacturing continuum, including discovery research, preclinical development, clinical trials, drug substance manufacturing, fill-and-finish, quality assurance, regulatory approval, and procurement or market access. Second, the Walt and Gilson policy triangle framework, comprising policy content, context, process and actors, will be used to analyse the policy architecture surrounding each stage of this continuum.

Based on available data, an analysis of policy coherence within and between countries will be done. For instance, how aligned are science policy, industrial policy and trade/IP policy in African countries? Similarly, an assessment of this policy coherence at national levels, regional economic community, and continental levels will be analysed and alignments and potential conflicts or gaps reported.

Both inductive and deductive coding approaches will be used. Coding will be conducted by a primary coder, and 25 percent of the coding will be reviewed by a secondary coder. Any disagreement between coders will be resolved by consensus or by discussion with a third coder.

Descriptive analysis will be used to synthesise and present the types and sources of documents included in this review. Data will be managed using Microsoft Excel 2021 for data charting. The extracted data will be imported into NVivo, which is qualitative data analysis software, to facilitate systematic data management and coding. NVivo will also be used for content and thematic analysis and synthesis of the coded data.

### Limitations

First, given the nature and objectives of a scoping review, it is not designed to determine the effectiveness of identified policies. However, where information is available, the review will describe key elements of policy content and implementation architecture, including policy objectives, financing mechanisms, implementation timelines, targets, coordination arrangements, and monitoring and evaluation plans. Second, no formal critical appraisal of the methodological quality of included policy documents, which is not mandatory for scoping reviews, will be undertaken. While this approach reflects the heterogeneity of eligible policy instruments, the authenticity and provenance of selected documents will be ascertained by retrieving them from verifiable databases and official websites. Finally, restricting this review to documents available in English may exclude relevant evidence from Francophone, Lusophone and Arabic-speaking African countries where English is not the official national language. The search will include documents issued by regional and continental bodies, where available in English, to partially mitigate this limitation. However, policies available exclusively in other languages may remain underrepresented. This restriction reflects the linguistic capacity and resources available to conduct consistent screening, extraction, and analysis and should therefore be considered when interpreting the findings of this review.

### Ethical consideration

This scoping review does not require ethical approval. A detailed manuscript detailing the results of the review will be published upon completion of the study.

## Conclusion

This scoping review protocol aligns with the JBI methodological guidance for conducting scoping reviews and will be reported in accordance with the PRISMA-ScR guidelines. The results of the continental mapping of policy architecture for vaccine R&D and manufacturing will contribute to the academic literature on vaccine security in Africa and, specifically, generate insights into policy-related gaps along the vaccine R&D-to-manufacturing continuum at national, regional and continental levels.

## Data (and Software) Availability

### Underlying data

There is no underlying data associated with this article.

### Extended data

Figshare: Scoping review protocol: Search strategy, data extraction instrument and PRISMA-ScR checklist. https://doi.org/10.6084/m9.figshare.31949979.v1 (Adenipekun 2026).

This project contains the following extended data:

• Appendix I: Pilot search strategy
• Appendix II: Database search strategy
• Appendix III: Data extraction instrument

Data are available under the terms of the Creative Commons Attribution 4.0 International license (CC-BY 4.0).

### Reporting guidelines

The PRISMA-ScR (Preferred Reporting Items for Systematic reviews and Meta-Analyses extension for Scoping Reviews), available in the extended data ( https://doi.org/10.6084/m9.figshare.31949979), will be used to report this study.
