# Supplementary material for: Mapping policy architecture for vaccine research, development and manufacturing in Africa: a scoping review protocol
Source: Open Res Afr. 2026 Oct 28;9:25. Originally published 2026 May 21. [Version 4] doi: 10.12688/openresafrica.16549.4 (PMC13768702; doi:10.12688/openresafrica.16549.4)
Supplement: Peer review history

# Reviewer report: Reviewer response for version 4

**DOI:** 10.21956/openresafrica.18223.r37003

**Published:** 2026-11-13

**Authors:** Chimdessa Fekadu Tsega

**Affiliations:** 1 Chinese University of Hong Kong, Hong Kong,, Hong Kong, Hong Kong

no further comments

Is the study design appropriate for the research question?

Yes

Is the rationale for, and objectives of, the study clearly described?

Yes

Are sufficient details of the methods provided to allow replication by others?

Partly

Are the datasets clearly presented in a useable and accessible format?

Yes

Reviewer Expertise:

Intellectual property, health policy, international trade

I confirm that I have read this submission and believe that I have an appropriate level of expertise to confirm that it is of an acceptable scientific standard.

# Reviewer report: Reviewer response for version 3

**DOI:** 10.21956/openresafrica.18198.r36670

**Published:** 2026-10-09

**Authors:** Jennifer Cole

**Affiliations:** 1 Royal Holloway, University of London, Egham, UK

Regarding my earlier comments on linking vaccine manufacture and hoarding to only HICs, I do not think this has been adequately addressed in paragraphs 1 and 2, as the term "and a small number of emerging economies" does not accurately describe the major emerging economies of China, India and Russia - who were all major producers of COVID19 vaccines. It would be more accurate to say, 'and major BRIC nations". Nor did all HICs have their own manufacturing capacity, as the current wording implies. Several LMICs, including Thailand, Malaysia, Cuba, had strong vaccine manufacturing capacity. See, e.g. [Ref 1]

 Middle Income counties such as China and India were major producers. 

 It is true that capacity was low in Africa, but this was not the case in other LMICs across the world. Please amend this with accurate information. 

 The severe limitation of including only policy instruments in English has not been adequately addressed as it does not make explicit the potential biases in this approach due to French influence on Francophone African countries, which has been evidenced in other literature, e.g. 

 [Ref 2]

 and 

 [Ref 3]

 Literature such as this needs to be referenced and a stronger rationale than convenience given for including only English language papers without sufficient consideration of the biases this will introduce to the results.

Is the study design appropriate for the research question?

Partly

Is the rationale for, and objectives of, the study clearly described?

Partly

Are sufficient details of the methods provided to allow replication by others?

Yes

Are the datasets clearly presented in a useable and accessible format?

Not applicable

Reviewer Expertise:

Health risk, vaccine acceptance, health communication, biosafety and biosecurity, critical health geopolitics

I confirm that I have read this submission and believe that I have an appropriate level of expertise to confirm that it is of an acceptable scientific standard, however I have significant reservations, as outlined above.

## References

[1] Vaccine manufacturing capacity in low– and middle income countries. *https://www.ucl.ac.uk/engineering/sites/engineering/files/vax-hub_policy_brief_long_version_vaccine_manufacturing_capacity_in_lmics.pdf* .10.2471/BLT.20.273375PMC824303234248217

[2] : The colonial effect: Language, trust and attitudes to science as predictors of vaccine hesitancy across Africa. *Cultures of Science* .2024;7(2) : 10.1177/2096608324125733898-118 10.1177/20966083241257338

[3] *https://www.taskforce.org/battling-low-covid-vaccination-rates-in-francophone-countries/* .

# Response

**Published:** 2026-10-09

**Authors:** Adebisi Adenipekun

**Affiliations:** University of Oxford Nuffield Department of Medicine, Oxford, England, UK

**RESPONSE –First comment**

 Thank you for this clarification. We agree that the wording could imply that vaccine manufacturing capacity was characteristic of HICs generally and did not clearly recognise the manufacturing capacity in major emerging economies and other LMICs. We have revised the Abstract and Introduction sections to distinguish countries with established vaccine manufacturing capacity from income-group classifications. The revised text acknowledges substantial manufacturing capacity in some HICs, major emerging economies including China, India and Russia, and several other middle-income countries, while noting that manufacturing capacity remained limited in Africa. This revision also addresses the previous framing, which could be interpreted as implying that all HICs possessed vaccine manufacturing capacity.  

**RESPONSE – Second comment**

 Thank you for emphasising this point and for providing additional references. We agree that restricting inclusion to policy instruments available in English introduces a potential language-related selection bias, particularly where national policy instruments from Francophone, Lusophone and Arabic-speaking African countries are not available in English. We have strengthened the Limitations section to make explicit that this restriction may result in more complete policy coverage for countries and institutions that routinely publish documents in English, while underrepresenting countries where relevant instruments are available exclusively in other languages. We have therefore clarified the implications of this potential bias for cross-country and regional comparisons. We acknowledge the literature cited by the reviewer concerning historical and linguistic influences on vaccine attitudes in African countries, including differences reported between French-speaking and non-French-speaking settings. However, this review concerns policy architecture for vaccine R&D and manufacturing rather than vaccine acceptance. The English-language restriction reflects the linguistic capacity and resources required to undertake consistent screening, extraction and analysis across the review, rather than an assumption that English-language evidence represents the entire continent. Official English-language versions of eligible policy instruments from non-Anglophone countries will remain eligible.

# Reviewer report: Reviewer response for version 2

**DOI:** 10.21956/openresafrica.18152.r36353

**Published:** 2026-09-29

**Authors:** Anderson Uchenna Amaechi

**Affiliations:** 1 University of Geneva, Geneva, Switzerland

Overall, there has been substantial improvement from the previous draft. 

 1. Scope creep from mapping to coherence assessment

 The research questions are generally clear and logically sequenced. However, the fourth question extends beyond policy mapping into policy coherence analysis. The authors may wish to provide more explicit criteria for determining alignment, misalignment, or coherence across policy levels to enhance methodological transparency and reproducibility.

 2. Language bias

 Restricting eligibility to English-language policy instruments may result in systematic underrepresentation of policy developments in several regions of Africa. You might want to add a limitation to this.

 3. Limited operationalization of "policy architecture"

 The paper defines policy architecture, but it remains somewhat abstract.

 Questions remain such as:

- What constitutes a strong policy architecture?
- How will policy architecture be distinguished from policy content?
- How will relationships among policy instruments be assessed?

While the concept of policy architecture is central to the review, further clarification of how it will be operationalized analytically would strengthen the protocol.

 4. Ambiguity between protocol and review

 While presented as a protocol, portions of the manuscript read more like a conceptual scoping review. Eg the manuscript alternates between describing planned methods and discussing anticipated analytical contributions, which at times blurs the distinction between a review protocol and a completed scoping review. The authors may wish to clarify the manuscript's primary purpose as a protocol and consistently use future-oriented language.

Is the study design appropriate for the research question?

Yes

Is the rationale for, and objectives of, the study clearly described?

Partly

Are sufficient details of the methods provided to allow replication by others?

No

Are the datasets clearly presented in a useable and accessible format?

Partly

Reviewer Expertise:

NA

I confirm that I have read this submission and believe that I have an appropriate level of expertise to confirm that it is of an acceptable scientific standard, however I have significant reservations, as outlined above.

# Response

**Published:** 2026-09-29

**Authors:** Adebisi Adenipekun

**Affiliations:** University of Oxford Nuffield Department of Medicine, Oxford, England, UK

1. Scope creep from mapping to coherence assessment

 The research questions are generally clear and logically sequenced. However, the fourth question extends beyond policy mapping into policy coherence analysis. The authors may wish to provide more explicit criteria for determining alignment, misalignment, or coherence across policy levels to enhance methodological transparency and reproducibility.

**RESPONSE - 1** Thank you for your review and observation. We agree that the fourth research question extends the review beyond descriptive policy mapping to a comparative assessment of policy coherence. This is intentional and aligns with the objective of the scoping review to identify, map and compare policy instruments across national, regional and continental levels. Policy coherence will be examined descriptively through the proposed content and thematic analysis of the included policy instruments, rather than applying an a priori normative or quantitative threshold to classify policy instruments as “coherent” or “incoherent”. In the Methodology section, we describe mapping policy instruments across the vaccine R&D-to-manufacturing continuum and then analysing them using the Walt and Gilson policy triangle, including policy content, context, process, and actors. The protocol also specifies that policy coherence will be examined within and between countries and across national, regional and continental levels, with alignments, potential conflicts and gaps reported. We therefore consider the existing analytical framework appropriate for the exploratory purpose of a scoping review protocol. More detailed analytical categories may need refinement during coding as the range and characteristics of the policy instruments become apparent, rather than imposing a potentially artificial definition of coherence before examining the evidence. We have therefore retained the current manuscript wording.

 2. Language bias

 Restricting eligibility to English-language policy instruments may result in systematic underrepresentation of policy developments in several regions of Africa. You might want to add a limitation to this.

**RESPONSE - 2** Thank you. We agree that restricting eligibility to policy instruments available in English may underrepresent relevant documents from Francophone, Lusophone and Arabic-speaking African countries. Where an official English version of policy instruments from these non-English-speaking African countries is available, it will be included. However, documents available only in languages other than English will be excluded, given the linguistic capacity and resources required to conduct consistent screening, extraction and analysis. We have updated the Limitation section in the latest version of the protocol to acknowledge the implications of the language restriction for the scoping review and its interpretation.

 3. Limited operationalization of "policy architecture"

 The paper defines policy architecture, but it remains somewhat abstract.

 Questions remain such as:

- What constitutes a strong policy architecture?
- How will policy architecture be distinguished from policy content?
- How will relationships among policy instruments be assessed?

While the concept of policy architecture is central to the review, further clarification of how it will be operationalized analytically would strengthen the protocol.

**RESPONSE - 3** Thank you. In this scoping review, policy architecture is not intended as a normative construct to classify policy systems as “strong” or “weak”, because assessing policy effectiveness or formally rating the quality of policy systems is outside the scope of this review. Rather, it serves as an organising concept for examining the configuration of policy instruments and the relationships among their content, actors, processes and institutional contexts across the vaccine R&D-to-manufacturing continuum. The protocol operationalises this in several ways by using policy instruments as the unit of analysis, stating that the data extraction process will capture policy content, context, process and actors, and describing the analysis process, including policy instrument mapping across stages of the vaccine R&D-to-manufacturing continuum and the analysis of horizontal and vertical policy coherence within countries and across national, regional and continental levels. Accordingly, policy content is one component of the broader policy architecture rather than synonymous with it. We believe the existing methodological description provides sufficient detail to operationalise the protocol while allowing the analytical categories to be refined inductively as the policy instruments are examined. We have therefore retained the current text.

 4. Ambiguity between protocol and review

 While presented as a protocol, portions of the manuscript read more like a conceptual scoping review. Eg the manuscript alternates between describing planned methods and discussing anticipated analytical contributions, which at times blurs the distinction between a review protocol and a completed scoping review. The authors may wish to clarify the manuscript's primary purpose as a protocol and consistently use future-oriented language.

**RESPONSE - 4** Thank you. We confirm that the manuscript is a scoping review protocol, not a report of a completed scoping review. It also states that we will publish a separate manuscript reporting the results once the review is complete. The variation in tense reflects the stage of the review process at the time of protocol preparation. Activities that had already occurred, such as the development and piloting of the search strategy, are appropriately reported in the past tense, whereas activities forming part of the substantive review, including screening, data extraction, coding, analysis, and presentation of findings, are described prospectively. Similarly, discussion of anticipated contributions is intended to explain the rationale and expected relevance of the proposed review rather than to report review findings. We therefore consider the distinction between completed protocol development activities and planned review activities to be appropriately represented in the manuscript and have retained the current wording.

# Reviewer report: Reviewer response for version 2

**DOI:** 10.21956/openresafrica.18152.r36380

**Published:** 2026-09-25

**Authors:** Jennifer Cole

**Affiliations:** 1 Royal Holloway, University of London, Egham, UK

This is an important and timely review which is likely to increase understanding of the current challenges facing Africa and help to inform future policy.

 However, there are two key challenges with how the protocol is currently planned, which could be amended to improve the robustness of the study:

 Firstly, an underpinning approach of the study is that HIC/Global North ‘hoard’ vaccines for their own populations, and it is implied that they widely banned vaccine exports during COVID. However, the only named example given of this is India – categorized as a LMIC at the time of the pandemic. This needs to be addressed.

 Furthermore, is there not a danger that R&D manufacture in Africa will itself be concentrated in more developed, emerging economies such as South Africa, Nigeria and Kenya, leaving smaller and poorer countries equally underserved? Will the papers also be screened for how equity across all African countries will be ensured?

 Secondly, the inclusion of only English language papers is problematic as more than 40% of Africa is Francophone, approx. 20% Arabic and only 40% have English as a major language (hardly any countries consider English as the official first language).

 This is problematic as France is one of the most vaccine hesitant countries in the world, and French connections, media, diaspora and other influence threatens to bias approaches to vaccine R&D and production in Francophone countries. An English-only inclusion criteria is therefore likely to bias the results.

 This needs to be critiqued in much more depth as a limitation of the study and, if possible, resources sought to address this through translation/translation software.

**Minor additional points are listed below:**

 In abstract:

 'geographically concentrated' where? Please list location. 

 Produce 60% of vaccine consumed locally - what is the current level, and therefore how much of an increase is needed? Figures in the main body of the text suggest that around 4% of locally consumed vaccines are manufactured in Africa at present. Please add this to the abstract.

 In introduction:

 Paragraph 2, line 2: again, define where the vaccine manufacture is 'geographically concentrated'.

 Paragraph 2, line 4: which countries ‘banned’ vaccine exports? It is correct to say that manufacturing countries protected vaccines for their own populations, but I am only aware of India (considered an LMIC itself at the time) officially banning exports.

 Paragraph 3, line 2: the statement “more vaccines than needed” does not seem to be supported by the paper cited and needs qualification. It may be correct that HICs vaccinated their own lower-risk populations before releasing vaccines to higher-risk groups in LMICs or provided their own populations with 2-3 does before releasing vaccines that could have provided first doses for populations elsewhere but this is different to stockpiling vaccines without using them, which is what the current phrasing implies. Please reword.

 Also, cf above – India is neither a HIC or a Global North country, so to use it as an example of Global North ‘hoarding’ is incorrect. Ensuring local manufacture offers protection for local citizens – which is what India was doing with this policy – is exactly what the authors are proposing for Africa.

 This needs to be reflected on rather than simply demonising the countries that acted this way.

 Paragraph 4, final line: the model could work for LMIC and LICs if local manufacture can be developed. This qualification needs to be added, particularly as it is the position the paper is supporting to introduce.

 In methodology:

 Protocol extraction: How will ‘documents emanating from Africa’ be determined? Does the responsible organization have to have a postal/head office address in an Africa country?

 What is the justification of only including documents in English considering the large number of francophone African countries and countries where policy documents may be written in Arabic? Could this bias the results at all? This is particularly problematic considering the large number of Francophone African countries and the high levels of vaccine hesitancy in France which may influence countries’ policies through French language media and diaspora populations in France. I would suggest this is either reconsidered or more consideration is given to the biases this might introduce, such as an unequal positioning of vaccine R&D and manufacture in Anglophone countries.

 Data analysis and presentation, line 1: explain ‘policy triangle framework’. Is this the health policy triangle (content/context/process influencing action)? Do not assume the readers will know this. Paragraph 3: How is the Walt & Gibson framework different from what has been described above?

 Limitations: Including English language papers only is given as a limitation of the study but still no rationale is given for it. Considering the large number of African countries where English is not the first language, this is likely to bias the results, particularly considering the potential influence of French-language media and African diaspora in France on Francophone African countries –France has one of the lowest rates of vaccine acceptance in the world.

 I would suggest the authors reconsider this exclusion and consider the use of translators or translation software to address this. If this is not possible, the impact of the limitation needs to be further considered and reflected on.

 Will particular attention be given to how such R&D and manufacturing capacity be financed? It is very well to have grand ambitions to considerably increase vaccine R&D manufacture but are the ambitions realistic and practical? Ensuring this is captured in the search protocol would strengthen the value of the study.

Is the study design appropriate for the research question?

Partly

Is the rationale for, and objectives of, the study clearly described?

Partly

Are sufficient details of the methods provided to allow replication by others?

Yes

Are the datasets clearly presented in a useable and accessible format?

Not applicable

Reviewer Expertise:

Health risk, vaccine acceptance, health communication, biosafety and biosecurity, critical health geopolitics

I confirm that I have read this submission and believe that I have an appropriate level of expertise to confirm that it is of an acceptable scientific standard, however I have significant reservations, as outlined above.

# Response

**Published:** 2026-09-29

**Authors:** Adebisi Adenipekun

**Affiliations:** University of Oxford Nuffield Department of Medicine, Oxford, England, UK

**COMMENT 1** - Firstly, an underpinning approach of the study is that HIC/Global North ‘hoard’ vaccines for their own populations, and it is implied that they widely banned vaccine exports during COVID. However, the only named example given of this is India – categorized as a LMIC at the time of the pandemic. This needs to be addressed.

**RESPONSE** Thank you for your review and comment. We agree that the previous framing risked conflating different manifestations of vaccine nationalism and could be misinterpreted as portraying India as an HIC/Global North country. The paragraph has been revised to differentiate the disproportionate advance procurement and domestic prioritisation of vaccine supplies by HICs from the temporary restriction of vaccine exports. The revised text focuses on the concentration of early vaccine procurement and prioritisation of domestic access and the resulting implication for timely access in LMICs and LICs.

**COMMENT 2** - Furthermore, is there not a danger that R&D manufacture in Africa will itself be concentrated in more developed, emerging economies such as South Africa, Nigeria and Kenya, leaving smaller and poorer countries equally underserved? Will the papers also be screened for how equity across all African countries will be ensured?

**RESPONSE** Thank you for highlighting this point. Geographic concentration and intra-African equity are crucial. Through this review, we would identify policy provisions relevant to regional coordination, manufacturing distribution, procurement, access, financing, technology transfer and cross-country coherence. However, determining whether equitable access will ultimately be achieved is outside the scope of this review.

**COMMENT 3** - Secondly, the inclusion of only English language papers is problematic as more than 40% of Africa is Francophone, approx. 20% Arabic and only 40% have English as a major language (hardly any countries consider English as the official first language).

 This is problematic as France is one of the most vaccine hesitant countries in the world, and French connections, media, diaspora and other influence threatens to bias approaches to vaccine R&D and production in Francophone countries. An English-only inclusion criteria is therefore likely to bias the results.

 This needs to be critiqued in much more depth as a limitation of the study and, if possible, resources sought to address this through translation/translation software.

**RESPONSE** Thank you. We agree that restricting eligibility to policy instruments available in English may underrepresent relevant documents from Francophone, Lusophone and Arabic-speaking African countries. Where an official English version of policy instruments from these non-English-speaking African countries is available, it would be included. However, documents exclusively in languages other than English will be excluded because of the linguistic capacity and resources available to conduct consistent screening, extraction and analysis. We have updated the Limitation section to acknowledge the implications of the language restriction for the scoping review and its interpretation.

**Minor additional points are listed below:**

**COMMENT 4** - In abstract:

 'geographically concentrated' where? Please list location. 

**RESPONSE** Thank you. We agree that being more specific about where COVID-19 vaccine production was concentrated will provide more clarity and better context for this review. We have updated the Abstract and Introduction sections to include High-Income Countries (HICs) and some emerging manufacturing economies.

**COMMENT 5 -** Produce 60% of vaccine consumed locally - what is the current level, and therefore how much of an increase is needed? Figures in the main body of the text suggest that around 4% of locally consumed vaccines are manufactured in Africa at present. Please add this to the abstract.

**RESPONSE** Thank you. We have included the baseline as reported by the African Union and Africa CDC, with the vision of increasing local vaccine manufacturing in Africa from less than one percent to 60 percent by 2040.

**COMMENT 6** - In introduction:

 Paragraph 2, line 2: again, define where the vaccine manufacture is 'geographically concentrated'.

**RESPONSE** Thank you. We agree that being more specific about where COVID-19 vaccine production was concentrated will provide more clarity and better context for this review. We have updated the Abstract and Introduction sections to include High-Income Countries (HICs) and some emerging manufacturing economies.

**COMMENT 7 -** Paragraph 2, line 4: which countries ‘banned’ vaccine exports? It is correct to say that manufacturing countries protected vaccines for their own populations, but I am only aware of India (considered an LMIC itself at the time) officially banning exports.

**RESPONSE** Thank you for the clarification. We have revised the manuscript to clearly distinguish HICs’ disproportionate advance procurement and prioritisation of domestic vaccine access from the decision by some manufacturing countries and regions (USA, India, and the European Union) to temporarily ban vaccine exports. This wording also resolved the comments about India being presented as a HIC.

**COMMENT 8** - Paragraph 3, line 2: the statement “more vaccines than needed” does not seem to be supported by the paper cited and needs qualification. It may be correct that HICs vaccinated their own lower-risk populations before releasing vaccines to higher-risk groups in LMICs or provided their own populations with 2-3 does before releasing vaccines that could have provided first doses for populations elsewhere but this is different to stockpiling vaccines without using them, which is what the current phrasing implies. Please reword.

**RESPONSE** Thank you. We have revised the manuscript to clarify our point about disproportionate advance procurement and prioritisation of domestic populations by HICs as opposed to vaccine stockpiling. The revised framing reflects the unequal distribution of early vaccine supplies and the resulting delays in access for many LMICs and LICs.

**COMMENT 9 - **Also, cf above – India is neither a HIC or a Global North country, so to use it as an example of Global North ‘hoarding’ is incorrect. Ensuring local manufacture offers protection for local citizens – which is what India was doing with this policy – is exactly what the authors are proposing for Africa.

 This needs to be reflected on rather than simply demonising the countries that acted this way.

**RESPONSE** Thank you. We agree that the previous framing risked conflating different manifestations of vaccine nationalism and could be misinterpreted as portraying India as an HIC/Global North country. The paragraph has been revised to differentiate the disproportionate advance procurement and domestic prioritisation of vaccine supplies by HICs from the temporary restriction of vaccine exports. The revised text focuses on the concentration of early vaccine procurement and prioritisation of domestic access and the resulting implication for timely access in LMICs and LICs.

**COMMENT 10 - **Paragraph 4, final line: the model could work for LMIC and LICs if local manufacture can be developed. This qualification needs to be added, particularly as it is the position the paper is supporting to introduce.

**RESPONSE** Thank you. We agree that the model could work for LMICs and LICs if local manufacture can be developed. We have revised the manuscript to include this qualification.

 In methodology:

**COMMENT 11 - **Protocol extraction: How will ‘documents emanating from Africa’ be determined? Does the responsible organization have to have a postal/head office address in an Africa country?

**RESPONSE** Thank you. We have updated the Methodology section with the detailed geographic scope of the eligible primary sources, which are policy instruments issued or formally endorsed by African national governments, African regional economic communities, the African Union or its agencies, and other recognised African intergovernmental bodies. Policy instruments issued by non-African/international organisations will be eligible only where they establish an explicit policy commitment, governance arrangement, technology-transfer mechanism or financing mechanism applicable to vaccine R&D or manufacturing in Africa.

**COMMENT 12 – **Data analysis and presentation, line 1: explain ‘policy triangle framework’. Is this the health policy triangle (content/context/process influencing action)? Do not assume the readers will know this. Paragraph 3: How is the Walt & Gibson framework different from what has been described above?

**RESPONSE** Thank you. We have revised the manuscript to explicitly define the policy triangle as the Walt and Gilson policy triangle framework, comprising policy content, context, process and actors.

**COMMENT 13 - **What is the justification of only including documents in English considering the large number of francophone African countries and countries where policy documents may be written in Arabic? Could this bias the results at all? This is particularly problematic considering the large number of Francophone African countries and the high levels of vaccine hesitancy in France which may influence countries’ policies through French language media and diaspora populations in France. I would suggest this is either reconsidered or more consideration is given to the biases this might introduce, such as an unequal positioning of vaccine R&D and manufacture in Anglophone countries.

 Limitations: Including English language papers only is given as a limitation of the study but still no rationale is given for it. Considering the large number of African countries where English is not the first language, this is likely to bias the results, particularly considering the potential influence of French-language media and African diaspora in France on Francophone African countries –France has one of the lowest rates of vaccine acceptance in the world.

 I would suggest the authors reconsider this exclusion and consider the use of translators or translation software to address this. If this is not possible, the impact of the limitation needs to be further considered and reflected on.

**RESPONSE** Thank you. We agree that restricting eligibility to policy instruments available in English may underrepresent relevant documents from Francophone, Lusophone and Arabic-speaking African countries. Where an official English version of policy instruments from these non-English-speaking African countries is available, it would be included. However, documents exclusively in languages other than English will be excluded because of the linguistic capacity and resources available to conduct consistent screening, extraction and analysis. We have updated the Limitation section to acknowledge the implications of the language restriction for the scoping review and its interpretation.

**COMMENT 14 - **Will particular attention be given to how such R&D and manufacturing capacity be financed? It is very well to have grand ambitions to considerably increase vaccine R&D manufacture but are the ambitions realistic and practical? Ensuring this is captured in the search protocol would strengthen the value of the study.

**RESPONSE** Thank you for this comment. We agree that financing is a key component of the feasibility and sustainability of vaccine R&D and manufacturing ambitions. Financing provisions within the eligible policy instruments will be captured. Financing will also be examined alongside the responsible institutions, implementation mechanisms, targets, timelines, and monitoring and evaluation arrangements. The review also included relevant financing mechanisms established by external organisations that are applicable to vaccine R&D or manufacturing in Africa. However, while the review will map and compare financing commitments and mechanisms within the identified policy architecture, it is not an independent economic feasibility or cost-effectiveness assessment of proposed vaccine R&D and manufacturing investments.

# Reviewer report: Reviewer response for version 1

**DOI:** 10.21956/openresafrica.17739.r35205

**Published:** 2026-07-24

**Authors:** Chimdessa Fekadu Tsega

**Affiliations:** 1 Chinese University of Hong Kong, Hong Kong,, Hong Kong, Hong Kong

- The protocol is methodologically well grounded. However, the reliance on ad hoc Google searches of 55 national government websites raises reproducibility concerns. This is mainly because the protocol does not specify search dates, the number of results to be screened per country, or how results will be documented for auditability.

 - Additionally, using Google Translate for documents in Africa's many official and working languages introduces a real risk of mistranslating legal or technical policy terminology, and the protocol does not describe any verification step, such as consultation with a native speaker or bilingual reviewer, to catch translation errors that could distort data extraction.

 - There is also a tension between the stated absence of timeframe restrictions and the instruction to include only the latest edition currently in force for documents with multiple editions, since the latter effectively excludes superseded versions regardless of when they were issued. The protocol should clarify how these earlier editions will be handled, as omitting them entirely could obscure how national agendas evolved toward or away from the continental 2040 target.

Is the study design appropriate for the research question?

Yes

Is the rationale for, and objectives of, the study clearly described?

Yes

Are sufficient details of the methods provided to allow replication by others?

Partly

Are the datasets clearly presented in a useable and accessible format?

Yes

Reviewer Expertise:

Health policy, intellectual property, and trade law

I confirm that I have read this submission and believe that I have an appropriate level of expertise to confirm that it is of an acceptable scientific standard, however I have significant reservations, as outlined above.

# Response

**Published:** 2026-09-09

**Authors:** Adebisi Adenipekun

**Affiliations:** University of Oxford Nuffield Department of Medicine, Oxford, England, UK

Thank you for your thoughtful review and feedback.

**First comment** The Methodology section has been revised to improve the transparency and reproducibility of the national government website searches. The revised search procedures specify that search dates, the number of search results to be screened for each country, and the procedures for documenting the searches and screening decisions to ensure consistent application across countries.

**Second comment** The Methodology section has been revised to address concerns regarding the potential for mistranslation of legal and technical policy terminology. The eligibility criteria have been refined to include policy instruments available in English, thereby removing reliance on Google Translate for data extraction and interpretation. The potential exclusion of relevant policy instruments available only in other languages has also been acknowledged as a limitation of the review.

**Third comment** The Methodology section has been revised to clarify how current and superseded policy versions will be handled. The revised approach specifies the treatment of earlier policy versions to ensure that relevant changes in national policy priorities and their evolution over time are appropriately captured.

# Reviewer report: Reviewer response for version 1

**DOI:** 10.21956/openresafrica.17739.r34785

**Published:** 2026-07-03

**Authors:** Anderson Uchenna Amaechi

**Affiliations:** 1 University of Geneva, Geneva, Switzerland

This manuscript addresses a timely and policy-relevant topic: the alignment of African national, regional, and continental policies for local vaccine production. The topic is important, and the protocol has potential value for policymakers, regional institutions, and implementation partners. However, in its current form, the manuscript requires substantial clarification, sharpening of scope, and stronger methodological detail before it is ready for indexing. 

**Decision: Major revision**

 Section-by-section review

 1) Title / framing

 Current issue

 The title and framing suggest a broad review of “local vaccine production” policies, but the body of the manuscript shifts between vaccine production, R&D, manufacturing, policy ecosystem, and alignment with continental strategy.

 Suggested note

 Comment: Please align the title, objectives, and methods so that the manuscript consistently reflects the same core concept throughout. At present, the scope appears broader in some sections and narrower in others.

 2) Introduction: opening paragraphs

 Original

 “The COVID-19 pandemic revealed gaps in the efficiency and effectiveness of established norms governing global vaccine research and development (R&D), manufacturing, and supply chains serving global populations equitably.”

 Suggested track-change revision

 “The COVID-19 pandemic exposed structural weaknesses in global vaccine research and development (R&D), manufacturing, and supply arrangements, particularly with respect to equitable access across regions and income groups.”

 Original

 “Before the pandemic, vaccines were produced and supplied mainly from high-income countries and some emerging economies…”

 Suggested track-change revision

 “Before the pandemic, vaccine production was concentrated in high-income countries and a small number of emerging economies, despite substantial vaccine demand in low- and middle-income countries.” This improves flow and removes repetition.

 Original

 “Despite the record-breaking speed of developing and manufacturing COVID-19 vaccines, production capacity was limited, largely concentrated in a few high-income countries, and was unable to promptly and equitably meet vaccine demand worldwide.”

 Suggested track-change revision

 “Despite the unprecedented speed of COVID-19 vaccine development, manufacturing capacity remained geographically concentrated and insufficient to meet global demand promptly and equitably.”

 Original

 “The inadequate vaccine supply capacity, coupled with other factors such as local politics, triggered vaccine nationalism…”

 Suggested track-change revision

 “This constrained production capacity, together with domestic political pressures and market power asymmetries, contributed to vaccine nationalism…” “Local politics” is vague; and this suggested revision is more analytically precise.

**Major Comment 1: The manuscript must explicitly distinguish itself from existing related protocols**

 The manuscript currently presents the topic as if there is no existing protocol-level work in this space. That is not sustainable given the existence of Amaechi et al. See **Reference 1.**

**Required revision**

 Add a paragraph in the introduction or review rationale that:

- cites Amaechi et al.,
- acknowledges the relatedness of the two protocols,
- states clearly how this review differs in question, sources, unit of analysis, and analytical framework.

**Major Comment 2: The review question is still too broad and conceptually unstable**

 The manuscript says it will review **local vaccine production policies**, but then also includes:

- vaccine R&D,
- manufacturing,
- policy ecosystem,
- international organizations’ documents,
- bilateral partner countries’ documents,
- technology transfer articles,
- laws, regulations, policy white papers, reports, documents, and articles.

This creates an extremely broad concept with no clear boundary.

 The result may become a heterogeneous collection of texts that are impossible to compare rigorously.

**Required revision**

 The authors should define:

- What counts as a policy?
- What counts as local vaccine production?
- Does this include only human vaccines?
- Does it include:
  - R&D policy,
  - manufacturing policy,
  - industrial policy,
  - procurement policy,
  - regulatory harmonization,
  - skills/workforce policy,
  - technology transfer arrangements,
  - financing instruments,
  - IP policy?
- Are journal articles included only as secondary contextual literature, or are they units for extraction?

At present, the protocol reads like a hybrid between a policy mapping review, gray literature review, and conceptual landscape analysis.

**Major Comment 3: Eligibility criteria need operational detail**

 Important selection rules are not adequately defined.

**Specific concerns**

- “Articles on technology transfer or decentralised vaccine manufacturing in LMICs and LICs will be included only if they contain specific references to Africa…”
  This is too vague. What qualifies as “specific references”?
- “All languages recognised as official in African countries, which can be translated into English using Google Translate…”
  This is pragmatic, but the protocol should explain how translation quality will be checked, especially for legal and policy terminology.
- “Only the latest edition currently in force will be included”
   Sensible in policy terms, but then how will the authors assess policy evolution over time?
- The protocol includes international organizations and countries with bilateral ties to Africa.
   This risks introducing a huge number of external documents without a defined rationale for inclusion.

**Required revision**

 Provide a **decision rule table** for inclusion/exclusion with concrete examples.

**Major Comment 4: Search strategy is not reproducible enough**

 For a protocol, the search section should be more exact.

**Missing details**

- date limits for each database search,
- full Boolean strings in the main paper or appendix,
- Google search procedures,
- number of pages/screen results to be screened,
- whether country website searches will be standardized,
- how archived or inaccessible pages will be handled,
- whether legal databases or AU regional economic communities’ document repositories will be searched systematically.

Without a transparent search protocol, website searching may become subjective and non-reproducible.

**Required revision**

 Specify:

- search dates,
- search syntax,
- website search rules,
- source hierarchy,
- documentation procedures for gray literature searching.

**Major Comment 5: The analysis plan is underdeveloped**

 The manuscript promises to map, assess, and compare policy content and alignment. That is more than descriptive cataloguing. Yet the analysis section remains generic.

**Current problem**

 Statements such as:

- “We will code the qualitative data into relevant categories and frameworks”
- “We will use descriptive analysis” do not adequately explain how comparison and alignment assessment will be done.

**Required revision**

 The authors should specify:

- the analytic framework,
- whether coding will be inductive, deductive, or hybrid,
- whether there will be a predefined matrix for comparing national/regional/continental alignment,
- what “alignment” means operationally,
- how disagreement between coders will be resolved during coding (not only screening),
- whether inter-coder calibration will be assessed.

A protocol of this type would benefit from a clearly described coding framework (e.g., domains such as governance, financing, R&D, infrastructure, technology transfer, regulatory systems, workforce, procurement/market shaping, regional cooperation).

**Major Comment 6: The manuscript should explicitly address quality appraisal—or justify its absence**

 For scoping reviews, formal critical appraisal is often not required, but the decision should be stated explicitly and justified.

 Amaechi et al. explicitly note that, consistent with JBI guidance, critical appraisal will not be performed.

 This manuscript should do the same, especially because it includes policy documents and reports of varied origin and quality.

**Major Comment 7: Internal inconsistencies reduce credibility**

 Several inconsistencies weaken the manuscript:

**Examples**

- The author voice shifts between “I will focus” and “we will…”
- The review is described as concerning local vaccine production, but elsewhere the emphasis becomes R&D pillar, then policy ecosystem, then manufacturing, then technology transfer.
- The abstract-like summary at the start is more concise and cohesive than the full manuscript, suggesting the main text was not fully harmonized.

**Required revision**

 Perform a full consistency edit for:

- authorial voice,
- terminology,
- scope,
- purpose statements,
- outcome language.

**5) Minor comments**

**Minor Comment 1: Improve precision of background claims**

 Some claims in the introduction are broad and rely on somewhat rhetorical wording (e.g., “the world returns to normal”). Consider a more scholarly and less narrative tone.

**Minor Comment 2: Clarify document types**

 “Policy white papers, reports, documents, and articles” is too loose. Distinguish between:

- normative policy instruments,
- strategic plans,
- legal/regulatory texts,
- technical guidance,
- commentary/literature used only for context.

**Minor Comment 3: Explain choice of tools**

 Why NVivo and Excel? What exactly will each be used for?

**Minor Comment 4: Address feasibility**

 Searching 55 country websites, 5 regional blocks, AU institutions, WHO AFRO, and partner-country documents may not be feasible unless tightly standardized.

**Minor Comment 5: Tighten the conclusion**

 The conclusion currently repeats that ethics approval is not required and that a results manuscript will be published. This is standard. It would be more useful to briefly restate the anticipated policy relevance.

 This protocol addresses an important and timely policy issue, but it requires major revision before being suitable for indexing. The manuscript’s central concern is not that it is an exact duplicate of existing work, but rather that it sits very close to an already published protocol on African vaccine manufacturing readiness and does not currently acknowledge or distinguish itself from that work. The authors should explicitly cite related protocols, sharpen their review question and eligibility criteria, and substantially strengthen the reproducibility and analytical detail of the methods.

Is the study design appropriate for the research question?

Yes

Is the rationale for, and objectives of, the study clearly described?

Partly

Are sufficient details of the methods provided to allow replication by others?

No

Are the datasets clearly presented in a useable and accessible format?

Partly

Reviewer Expertise:

Scoping review and evidence synthesis, Evaluation, Immunisation programmes, Global Health

I confirm that I have read this submission and believe that I have an appropriate level of expertise to confirm that it is of an acceptable scientific standard, however I have significant reservations, as outlined above.

## References

[1] : Assessing the Readiness of Local Vaccine Manufacturing in African Countries: Protocol for a Scoping Review. *JMIR Research Protocols* .2025;14: 10.2196/81231 10.2196/81231 PMC1277575741435379

# Response

**Published:** 2026-09-09

**Authors:** Adebisi Adenipekun

**Affiliations:** University of Oxford Nuffield Department of Medicine, Oxford, England, UK

***Section-by-section review***

***1. Title/framing*** *Thank you for your thoughtful review and feedback. The manuscript has been revised to consistently use the term “vaccine R&D and manufacturing” throughout, thereby more clearly reflecting the intended scope of the study. In addition, the policy conceptual framework has been clarified to ensure greater consistency and alignment with the study’s focus.*

***2. Introduction: opening paragraphs*** *The suggested revision has been accepted and incorporated into the manuscript, as it more clearly articulates the structural weaknesses in global vaccine R&D, manufacturing, and supply arrangements, particularly in relation to equitable access across regions and income groups.* ***2b, c, and d*** The suggested tracked change was accepted and incorporated into the manuscript.

**Major Comment 1: The manuscript must explicitly distinguish itself from existing related protocols** A paragraph has been added to the introduction section to explicitly position this scoping review in relation to existing evidence, including the protocol by Amaechi et al. and the related work by Ataguba et al. The new paragraph acknowledges the relatedness of these studies while clarifying the distinct contribution of the present review, particularly with respect to its research question, evidence sources, unit of analysis and analytical framework.

**Major Comment 2: The review question is still too broad and conceptually unstable** The primary and secondary research questions have been refined to more clearly define the scope and conceptual boundaries of the review. An operational definition of “policy” has been provided, together with examples of the policy categories relevant to vaccine R&D and manufacturing. The scope of “local vaccine production” has also been clarified. In addition, the eligibility and role of different evidence sources have been clarified to distinguish the sources that constitute units of analysis from those used as contextual evidence.

**Major Comment 3: Eligibility criteria need operational detail** The eligibility criteria have been revised to provide clearer operational guidance for study and document selection. In addition, a decision rule table has been included, specifying the inclusion and exclusion criteria and providing concrete examples to support consistent application of the eligibility criteria during the screening process.

**Major Comment 4: Search strategy is not reproducible enough** The search strategy has been revised to improve transparency and reproducibility. Detailed search string and database-specific search strategies, including search dates have been provided in the Appendix. The supplementary search materials are deposited in Figshare, with the repository link provided in the manuscript in accordance with the journal editors’ guidance ( https://doi.org/10.6084/m9.figshare.31949979). The grey literature search procedures have also been expanded to clarify search rules, source hierarchy, and procedure for identifying, screening, and documenting relevant sources. These revisions provide a more standardised approach to searching organisational and country websites and recording the grey literature search process.

**Major Comment 5: The analysis plan is underdeveloped** The data analysis section has been substantially revised to provide a clearer analytical approach. The revised section specifies the analytic framework and coding approach, including how policy content will be categorised and compared. It also clarifies how policy “alignment” will be operationalised and assessed and describes the procedures for resolving conflicts during data coding and analysis.

**Major Comment 6: The manuscript should explicitly address quality appraisal—or justify its absence** The manuscript has been revised to explicitly state that the formal critical appraisal of the included evidence will not be undertaken, consistent with the methodological approach for scoping reviews. The rationale for this decision has been provided, and the implications of including policy documents of varying origin and quality have been acknowledged in the Limitation section.

**Major Comment 7: Internal inconsistencies reduce credibility** A full consistency edit of the manuscript has been undertaken. The authorial voice, terminology, scope, purpose statements and outcome language have been reviewed and harmonised throughout the manuscript. In particular, terminology relating to vaccine R&D and manufacturing has been standardised to ensure that the framing and scope remain consistent across all sections of the manuscript.

**Minor Comment 1: Improve precision of background claims** The relevant statements in the Introduction section have been revised to improve precision and adopt a more scholarly and less narrative tone. Broad or rhetorical wording has been replaced with more specific and academically appropriate language.

**Minor Comment 2: Clarify document types** The Methodology section has been revised to more clearly distinguish the types of documents eligible for inclusion. The revised criteria specify the relevant categories of policy and related documents. The role of commentary and other peer review literature used primarily for contextual purposes has also been clarified.

**Minor Comment 3: Explain choice of tools**

 This Methodology section has been revised to clarify the rationale for using NVivo and Excel and the specific function of each tool. NVivo will be used to support the organisation, coding, and qualitative analysis, while Excel will be used to structure and manage extracted data and support descriptive analysis.

**Minor Comment 4: Address feasibility** The search approach has been revised to address feasibility and to improve transparency and reproducibility. A standardised procedure for searching country, regional, continental, and international organisation websites has been specified, including clearer search parameters and documentation procedures to ensure that the proposed search can be implemented consistently across the identified sources.

**Minor Comment 5: Tighten the conclusion** The Conclusion has been revised, with standard procedural statements removed and replaced by a clear statement of anticipated policy relevance and the review's potential contribution to vaccine R&D and manufacturing policy in Africa.  
